# Protocol for perfusing human axillary lymph nodes *ex vivo* to study structure and function in real time

**DOI:** 10.1016/j.xpro.2025.103624

**Published:** 2025-02-05

**Authors:** Amy Llewellyn, Rachel Barrow-McGee, Julia Stevenson, Jasmine Gore, Kalnisha Naidoo

**Affiliations:** 1 Translational Pathology, Comprehensive Cancer Centre, School of Cancer and Pharmaceutical Sciences, King’s College London, London, UK; 2 Toby Robins Breast Cancer Now Research Centre, Breast Cancer Research Division, The Institute of Cancer Research, London, UK; 3 King’s Health Partners Cancer Biobank, Guy’s Comprehensive Cancer Centre, London, UK; 4 Department of Cellular Pathology, King’s College Hospital, London, UK

**Keywords:** Cancer, Immunology, Tissue Engineering

## Abstract

Lymph nodes regulate immunity and maintain fluid balance in health and disease. Here, we present a protocol that uses normothermic perfusion to sustain patient-derived lymph nodes *ex vivo* for up to 24 h to study their structure and function. We describe steps for setting up both thermoregulatory and perfusion circuits, cannulating human lymph nodes, and perfusion. This protocol can be used to study how human lymph nodes change in cancer and other diseases, and/or in response to perturbations, including drugs.

For complete details on the use and execution of this protocol, please refer to Barrow-McGee et al.^1^

## Highlights

• Instructions for perfusing human lymph nodes *ex vivo* to maintain viability for 24 h
• Steps to measure real-time flow rates, acid-base balance, and perfusion pressures
• Can be used to study lymph node structure and function in different disease states
• Potential to test drug responses and discover biomarkers in patient-derived tissue

## Abstract (editor highlights)

Publisher’s note: Undertaking any experimental protocol requires adherence to local institutional guidelines for laboratory safety and ethics.

## Graphical abstract

**Figure:**
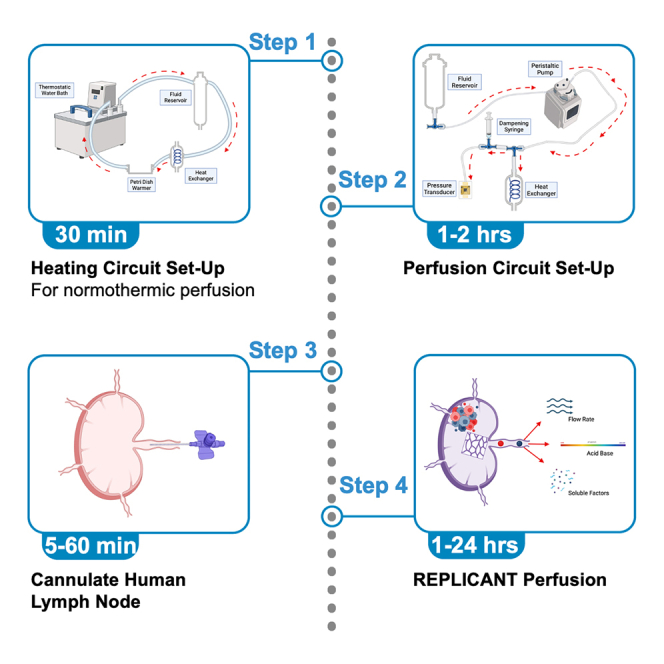

## Before you begin

**Timing: 1.5–3 h**

In breast cancer, axillary lymph nodes (ALN) play a crucial role in mediating adaptive immunity and regulating fluid balance, but they are also the first site of metastatic spread. However, we do not fully understand how breast cancer cells colonize ALN, compromising the host’s defenses. Bridging this knowledge gap is essential to determining when ALN should be treated or left in the patient to aid anti-tumor immune responses.

Ethically, accessing and obtaining human ALN tissue for research is difficult since the reporting pathologist has to examine every single node in its entirety to quantify disease burden.^2^ Consequently, most existing, patient-derived datasets largely have been obtained from tissue fixed at a specific disease stage.^3^^,^^4^^,^^5^^,^^6^^,^^7^^,^^8^ To address this, we developed the REPLICANT perfusion model, to facilitate real-time analysis of live, intact human ALN (structure and function) in a manner that does not adversely affect diagnosis and prognostication.^1^ This patient-derived model provides a unique opportunity to study ALN tissue biology in real time. Furthermore, we have shown that one can experimentally perturb the node during perfusion (for example, with drugs), and study how these perturbations alter the node. Both of these experimental designs make the model particularly relevant for biomarker discovery.^9^

Finally, while we have applied this protocol to study ALN harvested from breast cancer patients, this protocol could be applied to lymph nodes obtained from patients with other cancers and diseases.

**CRITICAL:** Obtaining appropriate ethical approval for using human lymph node tissue is essential. Protect patient confidentiality by de-identifying ALN samples and assigning unique study numbers to each patient, so that researchers remain blinded during analysis.

**Figure 1:**
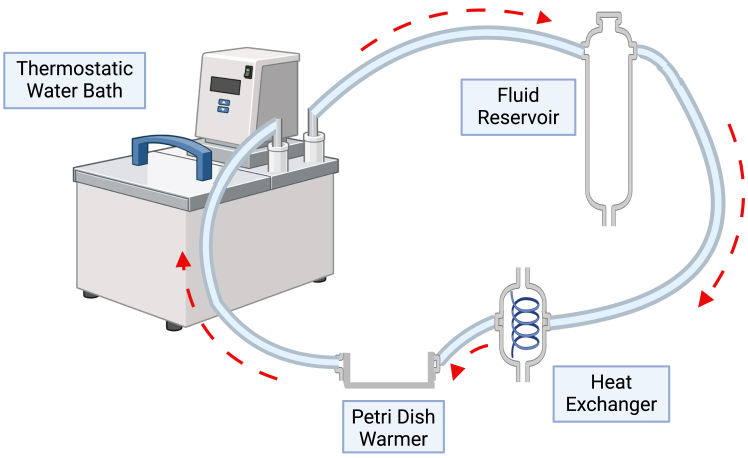
Connecting the thermoregulatory circuit Set up the water jacketed circuit so that warm distilled water flows in the direction of the arrows. The spiral heat exchanger should be positioned between the peristaltic pump and the cannulated axillary lymph node to compensate for any heat loss that occurs while Krebs-Henseleit solution transits through the pump head.

1. Prepare Krebs-Henseleit solution.
  a. Mix all the powders except calcium chloride, in 990 mL double distilled water (ddH_2_O) (see materials and equipment for details).
  b. Stir until fully dissolved (approximately 5 min).
  c. Add calcium chloride dihydrate diluted in 10 mL ddH_2_O slowly to the mixing solution.
  d. Filter using a disposable sterile bottle-top filter (0.22 μm membrane) within a tissue culture hood.
2. Set up the thermoregulatory (water jacketed) circuit (Figure 1) and pump distilled water through the jacketed glassware until it reaches 37°C.
  a. Connect the thermostatic water bath outlet to the glass reservoir inlet using 8 mm bore transparent PVC tubing.
  b. Connect the reservoir outlet to the spiral heat exchanger inlet.
  c. Connect the spiral heat exchanger outlet to the petri dish warmer inlet.
  d. Connect the petri dish warmer outlet to the inlet of the thermostatic water bath. Use retort stands to secure the glassware in position, ready for perfusion.

**CRITICAL:** Ensure that all connections are tightly screwed in to avoid any water leakage.

***Note:*** The glassware can be tilted once the water bath is switched on to remove any trapped gas bubbles.

**Figure 3:**
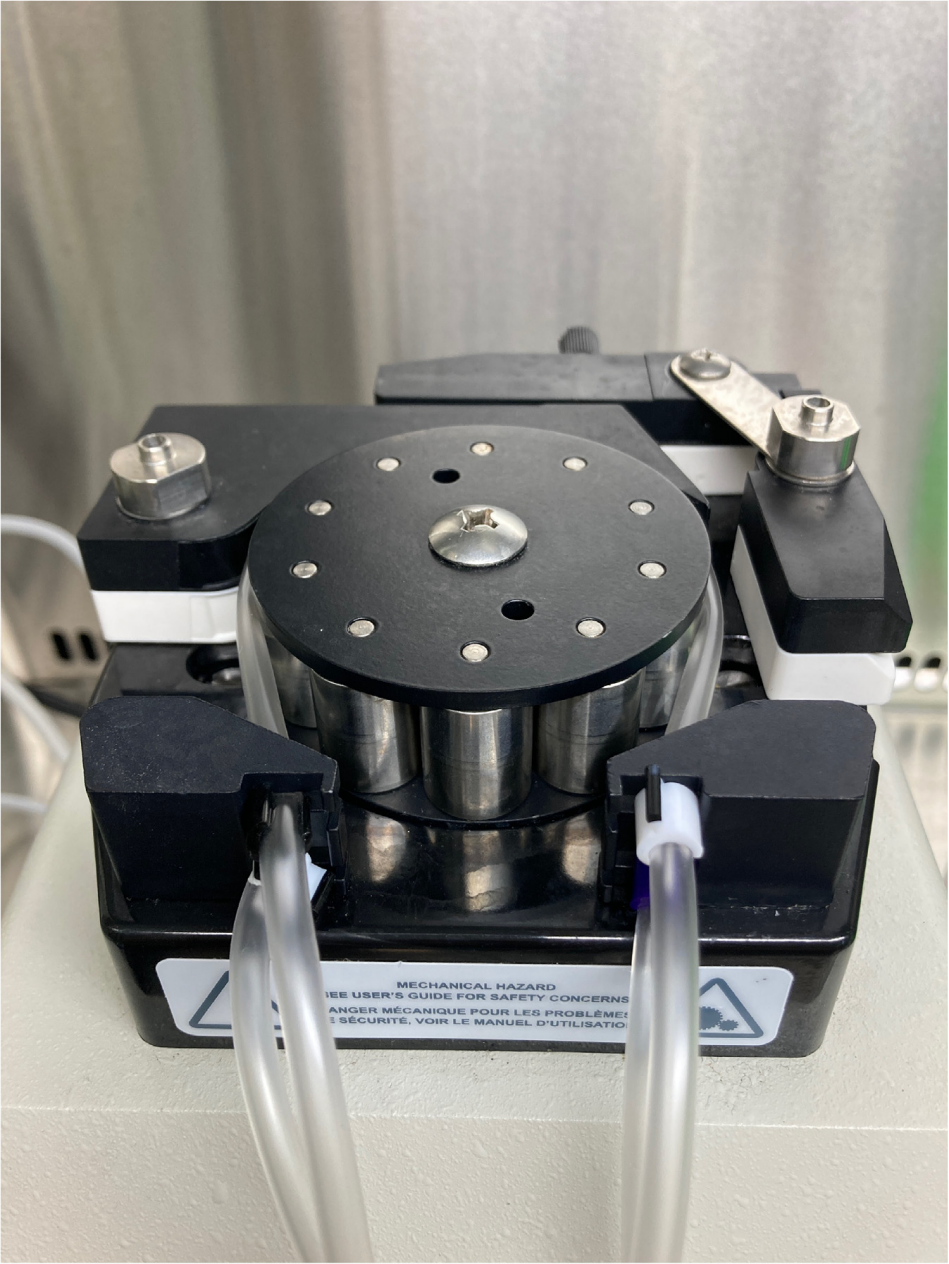
Photograph showing how Gilson tubing should be secured in the pump head
  b. Connect the glass reservoir to the purple tube in the peristaltic pump, using a stopcock.
  c. Split the fluid coming out of the pump (Figure 2) so that fluid can flow through the spiral heat exchanger (which warms it before it enters the ALN) into the cannulated ALN and simultaneously into the pressure transducer for the electrical feedback system to work.
  d. To improve the readout obtained, attach a damping syringe (half-filled with air) via a three-way stopcock to the line feeding the pressure transducer. This mechanically reduces any pressure oscillations that are produced by the peristaltic pump.
    ***Note:*** For accuracy, the pressure transducer should sit at the level of cannulated ALN artery.
    ***Note:*** Steps 1–3 can be performed the day before the perfusion experiment to save time if necessary.

3. Set up the perfusion circuit with an intravenous catheter attached at the end of the circuit to ensure that fluid flows from the glass reservoir into the ALN (Figure 2).
  ***Note:*** With regards to tubing, the following general principles apply: glass delivery lines fit 5 mm bore silicone tubing; the peristaltic pump requires specialized tubing (see below) that connects via connectors to 2 mm bore silicone tubing; the pressure transducer attaches to 2 mm bore silicone tubing; and three-way stopcocks allow one to transition between 5 mm and 2 mm bore tubing.
  a. Load the head of the Gilson Minipuls peristaltic pump with two sets of tubing: the first (2.06 mm internal diameter; purple in color) pumps fluid into the cannulated ALN; the second (3.18 mm internal diameter; black in color) pumps perfusate out of the petri dish into a collection bottle (single-pass perfusion) or back into the glass reservoir (recirculation). Ensure that both these tubes are taut and properly secured (Figure 3).

**Figure 2:**
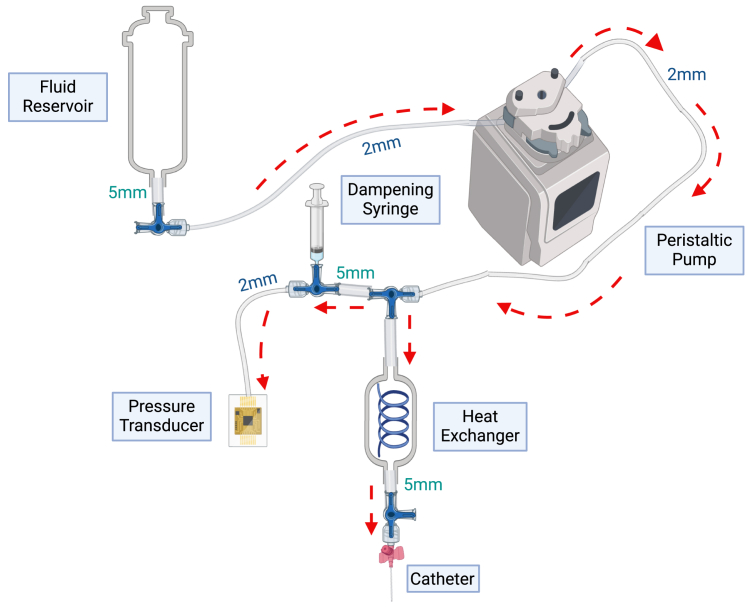
Connecting the perfusion circuit Set up the perfusion circuit so that Krebs-Henseleit solution flows in the direction of the arrows. This is achieved by using a combination of 5 mm bore tubing that fits the fluid delivery lines on the water jacketed glassware; 2 mm bore tubing that connects to the Gilson tubing and pressure transducer; and three-way stopcocks that connect these two different sizes of tubing. Of note, the circuit should split after exiting the peristaltic pump to carry fluid to both the pressure transducer and the cannulated axillary lymph node.
4. Set up the electrical circuit. We use the commercially available PowerLab 26 series digital data acquisition system (ADInstruments) to control and record ALN perfusion. There are three components to this system:
  a. Bridge Amp (single channel, non-isolated) which conditions (i.e., filters and amplifies) the signal from the pressure transducer for accurate measurement and control.
  b. STH Pump Controller which controls the peristaltic pump speed using a negative feedback electronic circuit.
  c. PowerLab which records data for downstream analysis.

**Figure 4:**
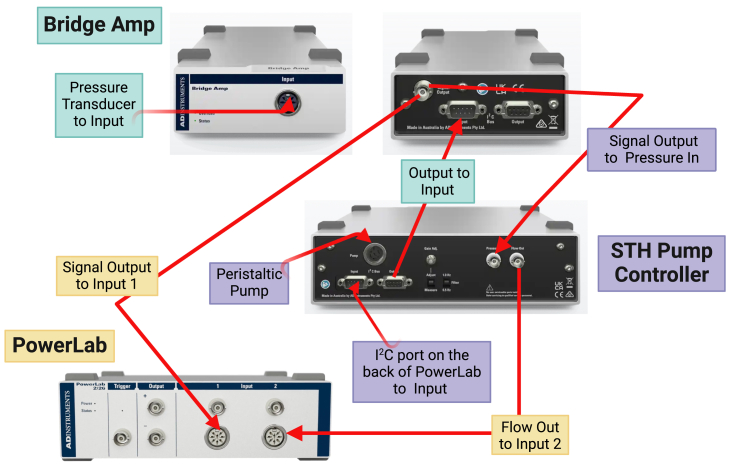
Schematic diagram illustrating how the electronic feedback system should be connected

Figure 4 shows how to connect these components. Support manuals and videos are also available on the manufacturer’s website.

5. Switch on the PowerLab system. This must always be switched on before the Gilson peristaltic pump to ensure that it can control the peristaltic pump, i.e., works correctly.
6. Switch on the Gilson peristaltic pump and set manually (i.e., using the buttons on the pump itself) to 48 rpm. This allows the electronic feedback system to set itself to the maximum possible flow rate.

***Note:*** If you find that switching the St Thomas’s Hospital (STH) Pump Controller on does not start the peristaltic pump, check first that the DIN cable is properly pushed in at the back of the Gilson Minipuls peristaltic pump. If this fails to correct the problem, switch off the PowerLab and the peristaltic pump and restart in the correct order.

7. Fill the glass reservoir with filtered Krebs-Henseleit solution.
8. Prime the perfusion circuit with Krebs-Henseleit solution.
  ***Note:*** You will need to prime both the line that leads to the catheter and the line that leads to the pressure transducer. This can be done by adjusting the three-way stopcocks on the circuit to direct fluid flow.
  Prime the line to the catheter first, and then prime the line to the pressure transducer as follows:
  a. Close the line leading to the pressure transducer.
  b. ‘Run’ the STH Pump Controller and prime the line leading to the catheter.
  c. Once the bubbles are removed, switch the STH Pump Controller off.
  d. Close the line leading to the catheter, and open the line leading to the pressure transducer.
  e. Squeeze the pressure transducer to allow fluid to run through it, preferably into a collection beaker/tube.
  f. ‘Run’ the STH Pump Controller and prime the line leading to the pressure transducer.
  g. Once the bubbles are removed, switch the STH Pump Controller off and stop squeezing the pressure transducer.
  h. Reset the stopcocks to allow flow in both directions.
    **CRITICAL:** Ensure that all bubbles are removed since this will affect the electrophysiological readouts (see, troubleshooting problem 1).
9. Calibrate pressure readings in the pressure channel in LabChart software using the two-point calibration function (detailed instructions on this are provided on the manufacturer’s website).
10. Calibrate flow readings in the flow channel in LabChart software using the two-point calibration function.

***Note:*** For inter-experimental reproducibility, we recommend selecting a certain speed (e.g., 17 rpm) and always calibrating the flow rate at that same speed for all experiments. By doing this, you will become familiar with what volume of fluid to expect at that speed during one minute of perfusion in your circuit, and as such, will be able to tell if the measurement is accurate or not. This will ensure that reproducible and comparable data is obtained across different experiments.

**Figure 5:**
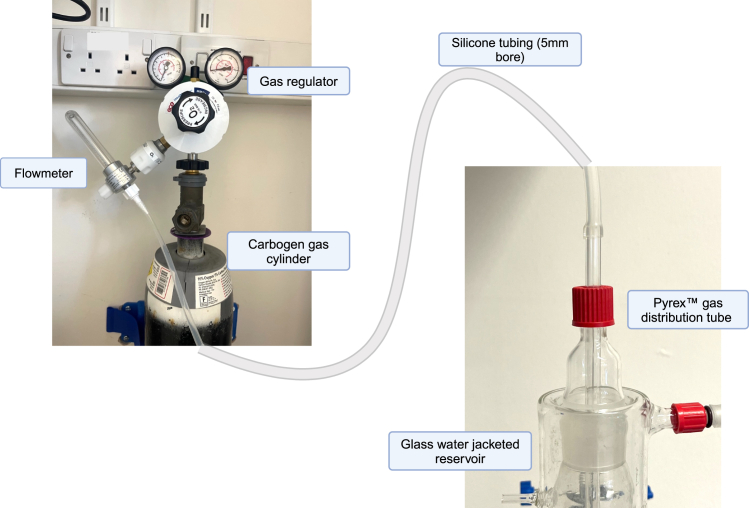
Schematic diagram, with photographs, showing how the gas supply should be connected

***Note:*** The bubbles in the Krebs-Henseleit solution in the glass reservoir are a good visual guide to the amount of gas being delivered to the circuit. Try to keep these bubbles at a gentle, steady state.

11. Deliver Carbogen gas (95% oxygen-5% carbon dioxide) to the perfusion circuit via a secure gas canister (Figure 5). Turn on the gas supply at least 15 min before the ALN is connected to the circuit.
  a. Attach a two-stage adjustable medical gas (oxygen) bull nose regulator including a Schrader valve to the canister.
  b. Attach a gas flow meter to the Schrader valve.
  c. Connect the gas flow meter to the glass gas distribution stick using 5 mm bore silicone tubing.
  d. Use a gas key and spanner combination tool to open the gas supply on the canister.
  e. Open the dial on the two-stage regulator – the dials should rise sequentially to indicate that gas is flowing through the regulator.
  f. Lower the gas distribution stick into the fluid in the glass reservoir during perfusion - this simulates a bubble oxygenator.
  g. Use the gas flow meter to control the amount of gas that enters the fluid in the glass reservoir.

12. Take an iStat reading once the fluid in the circuit has been gassed for at least 15 min to check that the pH is at 7.4.
13. Remove and discard the catheter that was used to prime the circuit, so that the final three-way stopcock on the circuit is ready to receive the cannulated ALN.

### Institutional permissions

All human ALN tissue samples used in this protocol were collected through clinical studies (i.e., Real-Time *Ex-Vivo* Perfusion of Lymph Nodes Invaded by Cancer with Novel Therapies (‘REPLICANT’) and the Breast Cancer Immune, Drug and Gene (‘BRIDGE’)) that were approved by the Health Research Authority via a Research Ethics Committee (REC) application (REPLICANT REC ref. 18/EE/0025; BRIDGE REC ref. 24/NW/0079). All patients provided informed consent for the use of their tissue in these studies. Others wishing to replicate this protocol will need approval from their respective funding agencies and/or institutions. Of note, the Breast Multidisciplinary Team treating the patient will have to agree that perfusing one or two ALN *ex vivo* for research purposes will not compromise patient care in any way. Furthermore, researchers will have to ensure that the ALN is repatriated to the diagnostic sample for inclusion in the final pathology report after perfusion.

## Key resources table

**Table:** 

REAGENT or RESOURCE	SOURCE	IDENTIFIER
**Biological samples**		

Human axillary lymph nodes	Patients under appropriate ethical consent	N/A

**Chemicals, peptides, and recombinant proteins**		

Sodium chloride (NaCl)	Sigma-Aldrich	Product Code: S5886
Sodium bicarbonate (NaHCO_3_)	Sigma-Aldrich	Product Code: S5761
Potassium chloride (KCl)	Sigma-Aldrich	Product Code: P5405
Magnesium sulfate heptahydrate (MgSO_4_.7H_2_O)	Sigma-Aldrich	Product Code: M2773
Sodium phosphate monobasic (NaH_2_PO_4_)	Sigma-Aldrich	Product Code: S5011
D-Glucose (C_6_H_12_O_6_)	Sigma-Aldrich	Product Code: G7021
Calcium chloride dihydrate (CaCl2.2H2O)	Sigma-Aldrich	Product Code: C7902

**Deposited data**		

Targeted gene expression data	Anonymized NanoString dataset of gene expression showing no significant changes in gene expression between the control ALN and those perfused using the REPLICANT system	https://zenodo.org/record/3543693
Perfusate proteome	Fluid circulating through the REPLICANT circuit during perfusion	https://www.ebi.ac.uk/pride/archive/projects/PXD022722

**Software and algorithms**		

LabChart software	ADInstruments	Available at: https://www.adinstruments.com/support/software

**Other**		

Lauda Alpha A6 heating thermostat	VWR	Cat#461-1204
Lauda Alpha A6 bath cover set	VWR	Cat#LAUDLCZE006
Water bath pump set	VWR	Cat#461-0054
MINIPULS 3 peristaltic pump with two channel pump head	Gilson	Cat#F155005
PowerLab 2/26	ADInstruments	Product Code: PL2602
Bridge Amp	ADInstruments	Product Code: FE221
STH pump controller	ADInstruments	Product Code: IN175
Disposable BP transducer	ADInstruments	Product Code: MLT0670
DIN (8) to MLT0670 cable (3.9 m)	ADInstruments	Product Code: MLAC06
Carbogen gas (95% oxygen – 5% carbon dioxide)	BOC	Part number: 131-F
Oxygen two-stage adjustable bullnose regulator including Schrader	MEC Medical Ltd	Part#6070SHA
Oxygen high flow 0–15 Lpm single flowmeter – fixed BS probe	MEC Medical Ltd	Part#6015DP1
Gas key and spanner combination tool	SLS (Scientific Laboratory Supplies)	Cat#GAS1008
i-STAT 1 analyzer	Abbott	Product Code: 04P7501
i-STAT CG4+ white cartridges	Abbott	Product Code: 03P85-25
Fisherbrand PVC transparent tubing	Fisher Scientific	Product Code: 12375859
AlteSil high strength silicone tubing 2 mm bore 1 mm wall	Altec	Product Code: 01- 93-1414
AlteSil high strength silicone tubing 5 mm bore 1.6 mm wall	Altec	Product Code: 01- 93-1428
PVC tubing ID 2.79 mm, 40 cm, 2 stops purple/white (12/PK)	Gilson	Product Code: F117948
PVC tubing ID 3.18 mm, 40 cm, 2 stops black/white (12/PK)	Gilson	Product Code: F117949
Connector, PVDF for 2–3 mm ID tubing to 2–3 mm, barbed, 3/32 × 3/32 (10/PK)	Gilson	Product Code: F1179951
Three-way stopcocks, Discofix	VWR	Cat#BRAU4095146
Pyrex gas distribution tube (porosity: 2)	Fisher Scientific	Product Code: 1560816
Glass water jacketed reservoir	Custom made	Blueprint shown in: Figure S1
Glass water jacketed spiral heat exchanger	Custom made	Blueprint shown in: Figure S2
Glass petri dish warmer	Custom made	Blueprint shown in: Figure S3
Retort stand sets complete kit with stand and 3 prong clamp	Camlab	Product Code: 1177157
Corning disposable sterile bottle-top filters, 0.22 μm membrane, 1000 mL	Corning	Product Code: 431174
Omron M2 Basic blood pressure monitor with 22–32 cm cuff	British Heart Foundation	Product Code: 6300090
Intravenous catheter 24 gauge, Surflo	VWR	Cat#TERUSR-OX2419C1
Intravenous catheter 26 gauge, Terumo	Medisave	Product Code: SR+DU2619PX
Ethicon MERSILK suture cutting needle: 16 mm, 75 cm black 5.0	Medisave	Product Code: W500H
Dissecting scissors, straight, 105 mm	Fisher Scientific	Product Code: 15207266
Blunt forceps, 115 mm	VWR	Cat#232-2112
Disposable syringes, 10 mL Luer slip, sterile	SLS	Cat# SYR1040
18G × 1.5″ blunt needle, Becton Dickinson	Camlab Limited	Cat#1203129
Corning non-treated culture dishes, D × H 100 × 20 mm	Sigma-Aldrich	Cat#CLS430591

## Materials and equipment

**Table:** 1 L Krebs-Henseleit solution

Reagent	Final concentration (mM)	Amount
Sodium Chloride (NaCl)	118.5	6.93 g
Sodium Bicarbonate (NaHCO_3_)	25	2.10 g
Potassium Chloride (KCl)	3	0.22 g
Magnesium Sulfate Heptahydrate (MgSO_4_.7H_2_O)	1.2	0.30 g
Sodium Phosphate Monobasic (NaH_2_PO_4_)	1.2	0.14 g
D-Glucose (C_6_H_12_O_6_)	11	1.98 g
Calcium Chloride Dihydrate (CaCl2.2H2O)	1.4	0.21 g
Double Distilled Water (ddH_2_O)	N/A	1000 mL
Carbogen gas (95% oxygen-5% carbon dioxide)	N/A	As required
**Total**	N/A	1000 mL

Krebs-Henselseit should be made fresh on the morning of the experiment and once made, can be stored at 19°C–24°C for up to 24 h.

## Step-by-step method details

### Harvesting and cannulating an ALN

**Timing: 5–60 min**

Dissect out the ALN from the surgical specimen and cannulate to allow attachment to the REPLICANT circuit.

**CRITICAL:** Aim to minimize cold ischemia time to preserve sample viability. Co-ordinate with the surgical team for immediate fresh tissue collection. Transport samples promptly to a designated cut-up area for ALN harvest and cannulation.

1. Palpate and dissect a fresh ALN out of the surgical specimen with its feeding vessels intact using blunt-end forceps at room temperature.
2. Cannulate the largest ALN feeding artery with either a 24- or 26- gauge intravenous catheter, and secure with a silk suture (Figure 6; see troubleshooting problem 2).

**Figure 6:**
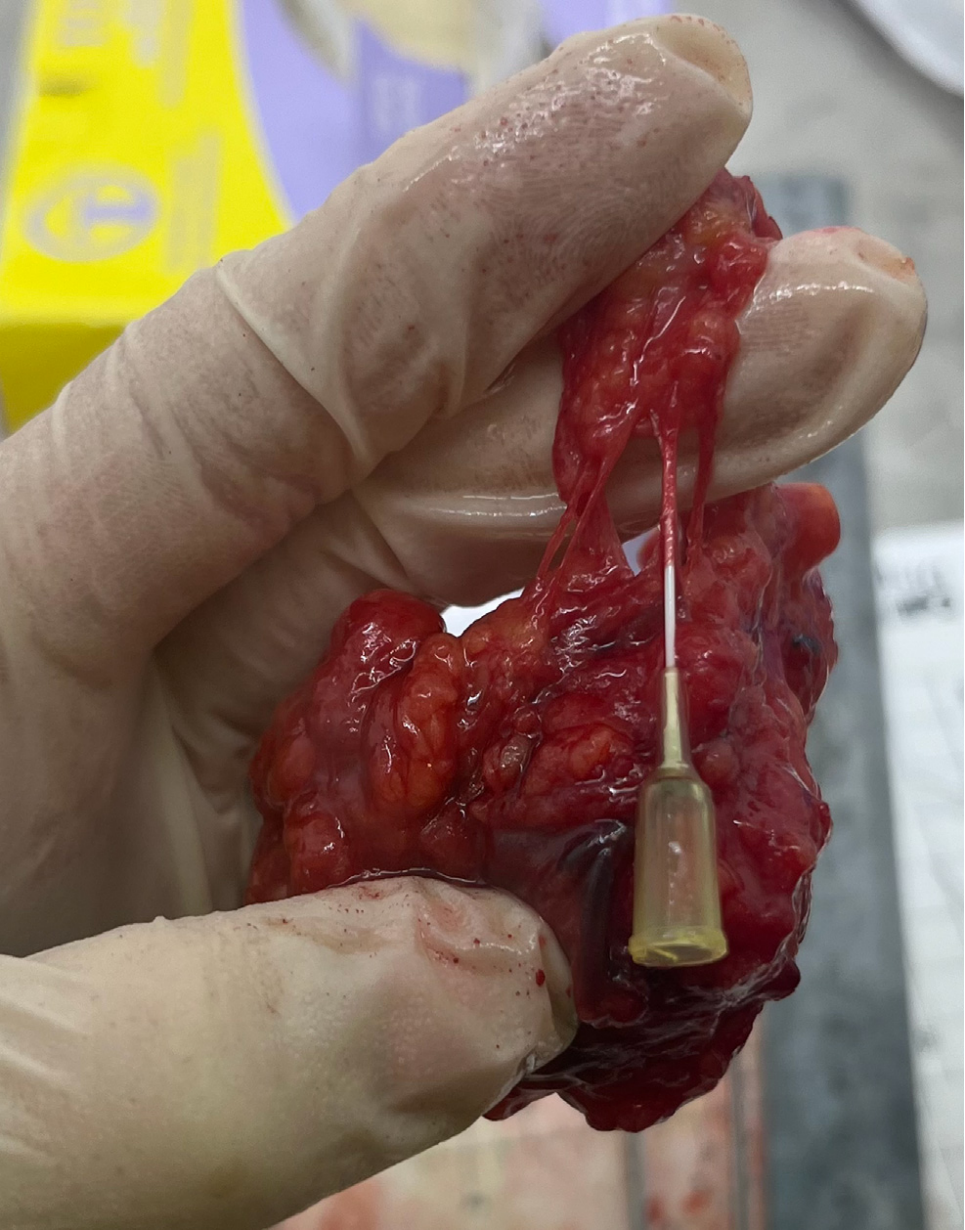
Photograph showing a cannulated axillary lymph node that has been secured with a silk suture
3. Cut the cannulated node free of the surrounding fatty tissue with dissecting scissors.
4. Place the cannulated ALN into a clean, sterile petri dish. The node is now ready to be attached to the perfusion circuit.

### REPLICANT perfusion of a patient-derived ALN

**Timing: 1–24 h**

Perfuse the ALN to obtain real-time data on flow rate, perfusion pressure and acid-base balance whilst collecting tissue and protein-rich perfusate for future analysis.

5. Gently screw the cannulated ALN into the three-way stopcock at the end of the circuit (see troubleshooting problem 3).
6. Start recording in LabChart software.
7. ‘Run’ the STH Pump Controller.
8. Watch the ALN to see when fluid seeps out the surface opposite the catheter. As soon as this happens, ‘Hold’ the STH Pump Controller.

***Note:*** Once the system holds, it ‘cuts out’ as it adjusts to the set pressure. This is quite alarming since the pump stops - it looks and sounds as if there has been an electrical failure. Don’t panic; it can take a minute for the circuit to restart. If the pump has not restarted after approximately 90 s, check that the feeding artery of the ALN isn’t kinked. Gently adjusting the position of the ALN in the petri dish will usually restart perfusion.

9. Use the ‘Adjust’ dial on the STH Pump Controller to reach a perfusion pressure of 40 mmHg.
10. We recommend that you use single-pass perfusion (Video S1) for the first hour, to pump out the blood and soluble factors that have accumulated in the node. Thereafter, either single-pass perfusion or reperfusion can be used, depending on the experimental set-up.

***Optional:*** You can collect and freeze down this perfusate for downstream analysis if you wish (this applies to later time-points as well).

11. Once the ALN has settled, gently place the petri dish lid over the sample. This helps maintain sterility and can be removed when iStat readings need to be taken.

***Note:*** You may need to cut openings in the plastic petri dish to allow the three-way tap and catheter. The petri dish lid should also create a humidified atmosphere for the ALN – beads of fluid will collect on it as the ALN is perfused. This is a reassuring ‘check’ that all is well during the experiment.

12. Take an iStat reading at 1 h to check the acid-base status of the node. We recommend taking regular readings at 4-hourly intervals thereafter for the duration of the experiment.
13. At the end of the experiment, flick the ‘Run’ switch off on the STH Pump Controller to stop perfusing and stop recording in LabChart.
14. Gently unscrew the ALN from the catheter and transfer immediately into formalin for fixation. Ensure that this node is repatriated to the rest of the diagnostic sample for routine pathological reporting.
15. The LabChart data can be exported as a raw text file and transferred to your preferred data analysis software (see troubleshooting problem 5).

Video S1. REPLICANT perfusion of a human axillary lymph node, related to step 10

### Disassembling the circuit

**Timing: 45–60 min**

Drain the contents of both the perfusion and thermoregulatory circuits and disconnect the various components. Discard all the tubing from the perfusion circuit and autoclave all glassware in preparation for the next experiment.

16. Switch off the gas supply and the circulating water bath.
17. Pump any remaining Krebs-Henseleit solution out of the perfusion circuit.
18. Drain the distilled water out of the water-jacketed circuit.
  a. Disconnect the hose attached to the water bath outlet and raise it (the height creates pressure that will force the fluid in the pipes to drain toward the inlet).
  b. Systematically drain water from the pipes and jacketed glassware in sequence until all the fluid has returned to the water bath.
19. Disconnect and discard all the tubing, connectors and three-way stopcocks from the perfusion circuit.
20. Disconnect the jacketed glassware. Keep the tubing aside for the next experiment.
21. Wrap all glassware generously in foil to protect the delicate parts and autoclave in between experiments to maintain sterility.

## Expected outcomes

REPLICANT perfusion can sustain human ALN for up to 24 h *ex vivo*.^1^ Due to diagnostic constraints, viability assessments on the tissue itself are limited to microscopy (necrosis and/or apoptosis), immunohistochemistry (Ki67) and targeted gene expression (available at https://zenodo.org/record/3543693). We have shown that perfusion did not significantly alter these parameters when compared to matched baseline control ALN that were fixed at time-point zero.^1^

Using this protocol, one can either perform single-pass perfusion or recirculate the perfusate.

In single-pass perfusion, the fluid exiting the ALN is removed from the circuit immediately. This fluid, which has only permeated through the ALN once, can be collected and analyzed downstream. We have previously used this method to administer drugs through the circuit at an appropriate dosage (i.e., dependent on tissue weight) to ALN.^1^ Using the monoclonal antibodies Trastuzumab and Nivolumab as chemical probes,^10^ we were able to show that flow was maintained to the entire ALN during perfusion.^1^ While Trastuzumab bound to HER2-positive cancer cells within the node without inducing necrosis, Nivolumab, a Programmed Death- Ligand 1 (PD-L1) checkpoint inhibitor, induced necrosis in a metastatic ALN from a patient with triple-negative breast cancer. Thus, drug effect can be monitored by quantifying necrosis and/or apoptosis in the perfused ALN tissue. In addition, the flow rate increased significantly during Nivolumab administration to the node. We therefore believe that flow rate can be used to monitor drug response during perfusion, but we are still validating this in a larger cohort of patient samples.

During recirculation on the other hand, the perfusate exiting the ALN is peristaltically pumped back into the glass reservoir and re-enters the ALN multiple times. This establishes a ‘feedback loop’ where the effects of ALN-secreted soluble factors can be tested and monitored.

We routinely monitor perfusate pH and lactate levels during perfusion with an iStat analyzer. Acid-base balance should remain stable over the 24-h perfusion period; any increase in lactate serves as an ‘alert’ that tissue viability might be compromised. Being able to monitor changes in flow rate in real time, at a resolution of 0.1 mL/min, is one of the most exciting features of this system. Interestingly, metastatic ALN had significantly higher flow rates than non-cancerous ALN.^1^ This increase started 2 h into perfusion, plateaued at 8 h and remained consistently elevated for the duration of the experiment. We are currently investigating the reasons behind this, but it does suggest that flow rate can be used to infer if metastasis is present in the node or not.

Finally, we showed that the perfusate is protein-rich and contains potential biomarkers of metastasis.^9^

Using shotgun proteomics, we identified 1453 proteins within ALN perfusate. Of these, 119 were significantly differentially expressed between the non-cancerous and metastatic perfused ALN.^9^ Pathway analysis of these 119 proteins showed that in metastatic nodes, immune function was diminished in favor of ‘extracellular matrix degradation’; only ‘neutrophil degranulation’ was preserved. We are currently validating two potential biomarkers identified through this work in a larger series of patient samples.

In summary, REPLICANT perfusion allows one to monitor how human ALN structure and function change in different disease states (in our case, non-cancerous versus metastatic ALN) or in response to perturbations, e.g., drugs. Structural changes can be monitored by comparing baseline, non-perfused and perfused nodes using histology with ancillary tests (e.g., immunohistochemistry). This novel model also facilitates functional analysis by coupling real-time measurements (acid-base balance and flow rate) with downstream multi-omics. Although we have chosen to use this system to investigate breast cancer metastasis, it could be applied to ALN obtained from patients with other cancers or benign diseases affecting the lymph nodes. For example, since each perfused ALN mimics the immune response of each patient *ex vivo*, REPLICANT perfusion could be used to test how specific cytokines induce tissue damage and alter immune function in patients with autoimmune diseases. This could even be tested at different flow rates or using drugs as blocking agents to unpick mechanism.

## Limitations

A current limitation of the model is that one cannot be definitively sure of the disease status of an ALN before perfusion. Whilst some large macrometastases (>2 mm in size) may be palpable, smaller tumor deposits are not. This should be taken into consideration when designing the experiment. Furthermore, all patient-derived ALN must be formalin-fixed and paraffin wax embedded after perfusion and repatriated to the diagnostic sample for pathological reporting. This limits the types of analysis that one can perform on the tissue itself. Nothing that could significantly harm/damage the tissue can be administered into the circuit during perfusion, nor can the tissue be digested for single cell characterization or metabolic analysis at the end of perfusion. Despite this however, we have shown, as outlined above, that combining the real-time perfusion data with perfusate analysis and/or subsequent examination of the formalin-fixed ALN tissue yields structural and functional data.^1^^,^^9^

## Troubleshooting

### Problem 1

Persistence of gas bubbles within the perfusion circuit after priming (before you begin).

### Potential solution

Care must be taken to remove all gas bubbles within the perfusion circuit before calibrating and attaching the lymph node. Bubbles of gas within the circuit will lead to artefactual sudden peaks in the electrophysiological readouts and have the potential to damage the lymph node. We recommend increasing the flow rate whilst priming and moving through the circuit in a systematic fashion, tilting the glassware upwards to remove bubbles. When tilting the glassware, cover the catheter with tissue paper to prevent water sprays and remain dry.

### Problem 2

Difficulty cannulating the feeding artery of the lymph node (Step 2).

### Potential solution

For ALN harvest and cannulation, a trained medical professional (usually a surgeon or pathologist) is required. We strongly advise that this trained professional practice either on fresh, postmortem or formalin-fixed tissue until proficient.

In our experience, it is best to start by palpating the specimen to find the largest lymph node and then dissect away some surrounding fat using two pairs of blunt-ended forceps into order to expose the vessels. After an appropriate vessel has been identified, hold the lymph node in your non-dominant hand, applying gentle traction to the vessel and cannulate. Once cannulated, secure in place with a silk suture before cutting the node and feeding vessel free from the surrounding fat.

The average size of the ALN feeding arteries varies. In all our experiments, we have opted to perfuse ALN that can be cannulated with a 24- or 26- gauge intravenous catheter. It might be possible to cannulate smaller ALN feeding arteries with specialized, custom-made catheters if needed, but we have never tried this.

Minimizing cold ischemia time is essential to ensure that the lymph nodes remain viable during perfusion. It is preferable to place the cannulated ALN onto the perfusion circuit within 20 min, but we have managed to successfully perfuse ALN that have taken as long as 60 min to dissect out and cannulate.

### Problem 3

Difficulty connecting the cannulated lymph node to the REPLICANT perfusion circuit (Step 5).

### Potential solution

Connecting the node to the circuit is technically challenging. Be gentle and take your time - it often takes practice to get this step right. If the artery has been punctured during cannulation, this will cause the vessel itself to swell and fluid will not reach the ALN. If the puncture is high up, you can sometimes bypass this and still perfuse successfully. If not, it unfortunately will mean the experiment will have to be terminated.

### Problem 4

Feeding artery slipping down the catheter during perfusion (Step 8).

### Potential solution

When setting up the circuit, keep the angle between the heat exchanger and the Petri dish containing the cannulated lymph node as acute as possible. If the angle is too obtuse, the combination of gravity and fluid flow will cause the lymph node to slowly slip down and eventually off the catheter.

### Problem 5

Difficulty exporting perfusion pressure and flow rate data for analysis (Step 15).

### Potential solution

When exporting raw data as text from LabChart, the data will include perfusion pressures and flow rates measured at 0.025 s intervals. For long experiments (e.g., several hours), this results in a large dataset, making it difficult to analyze trends over minutes or hours. To address this, we recommend “downsampling” the data to reduce its size. For instance, downsampling by 10 provides 0.25 s intervals, by 100 provides 2.5 s intervals, and by 2,400 provides one measurement per minute. Choose a downsampling rate based on the precision required for your experiment.

Steps to downsample data in LabChart:

• Go to File → Export.
• Name your file and ensure it is saved as a “LabChart Text File”.
• In the export settings, check the “Downsample by” box and input your desired downsampling value.

## Resource availability

### Lead contact

Further information and requests for resources and reagents should be directed to and will be fulfilled by the lead contact, Dr Kalnisha Naidoo, kalnisha.1.naidoo@kcl.ac.uk.

### Technical contact

Technical questions on executing this protocol should be directed to and will be answered by the technical contact, Dr Amy Llewellyn, amy.2.llewellyn@kcl.ac.uk.

### Materials availability

This study did not generate new unique reagents.

### Data and code availability

The targeted gene expression data generated during this study are available at Zenodo: https://zenodo.org/record/3543693. The proteomic data generated during this study are available at PRIDE: https://identifiers.org/pride.project:PXD022722.

## Acknowledgments

We would like to thank the patients for consenting to the use of their tissue to develop this model. This work was funded by Cancer Research UK (Pioneer Award CRC598X and supported by the 10.13039/501100000289CRUK City of London Centre Award CTRQQR-2021\100004) and Breast Cancer Now (Dame Vera Lynn Breast Cancer Now Clinical Research Training Fellowship 2023.06CRTF1643). We would like to thank Professor Michael J Shattock for providing advice on setting up the perfusion circuit and electronic feedback system. The graphical abstract/figures were created using Biorender.com.

“For Prishani, who always encouraged me to see the (im)possible.” – Kalnisha.

## Author contributions

K.N. conceived the protocol; K.N., R.B.-M., and A.L. designed the experiments; A.L., R.B.-M., J.S., and K.N. conducted the experiments and interpreted the data; A.L., J.G., and K.N. troubleshot and refined the protocol; A.L., R.B.-M., J.S., J.G., and K.N. wrote and edited the manuscript.

## Declaration of interests

The authors declare no competing interests.
