# Supplementary material for: Protocol for perfusing human axillary lymph nodes ex vivo to study structure and function in real time
Source: STAR Protoc. 2025 Feb 5;6(1):103624. doi: 10.1016/j.xpro.2025.103624 (PMC11848449; doi:10.1016/j.xpro.2025.103624)
Supplement: Document S1. Figures S1–S3 [file mmc1.pdf]

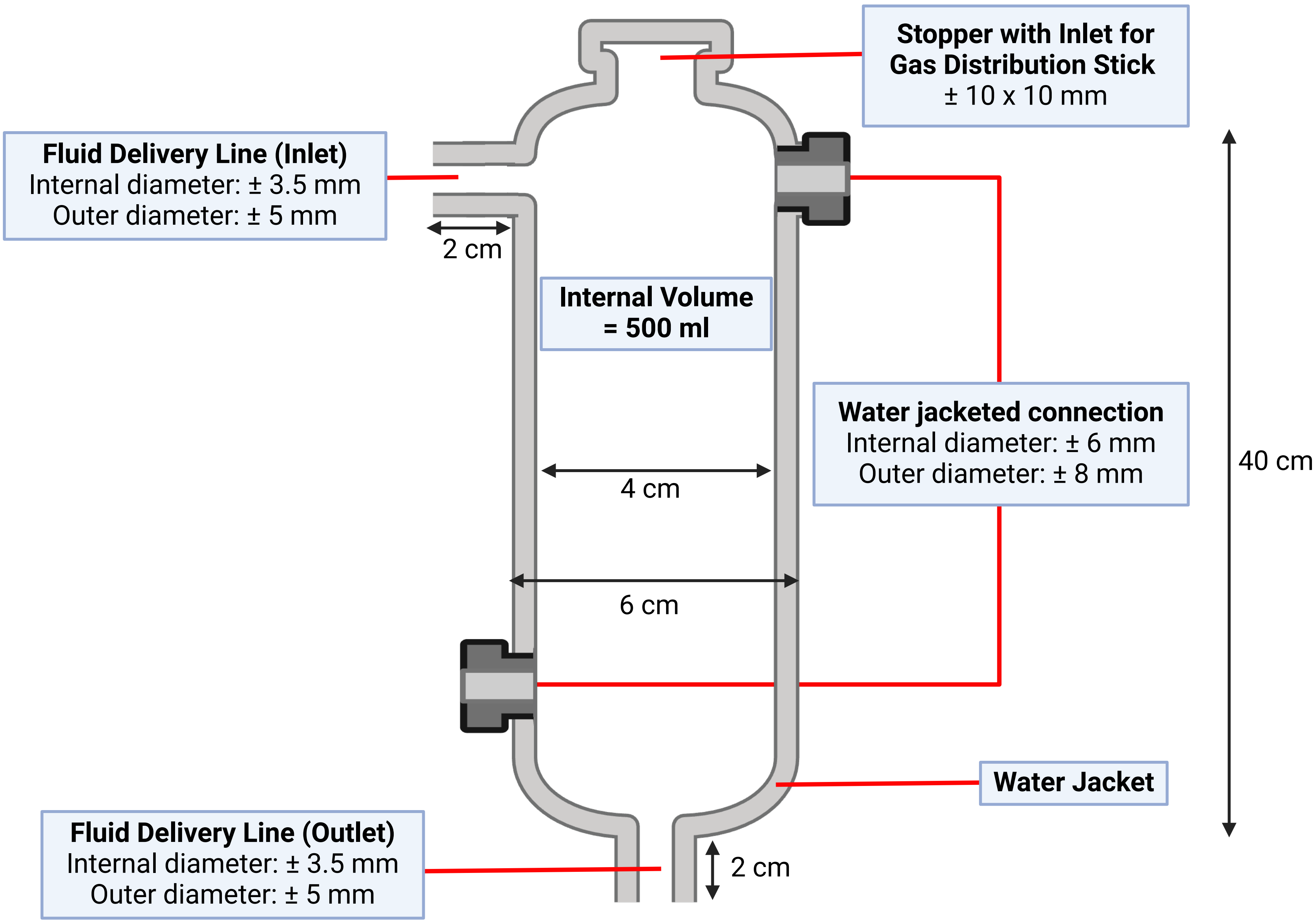

Supplementary Figure S1 Blueprint for a water jacketed glass reservoir (not drawn to scale; related to Key Resources Table).

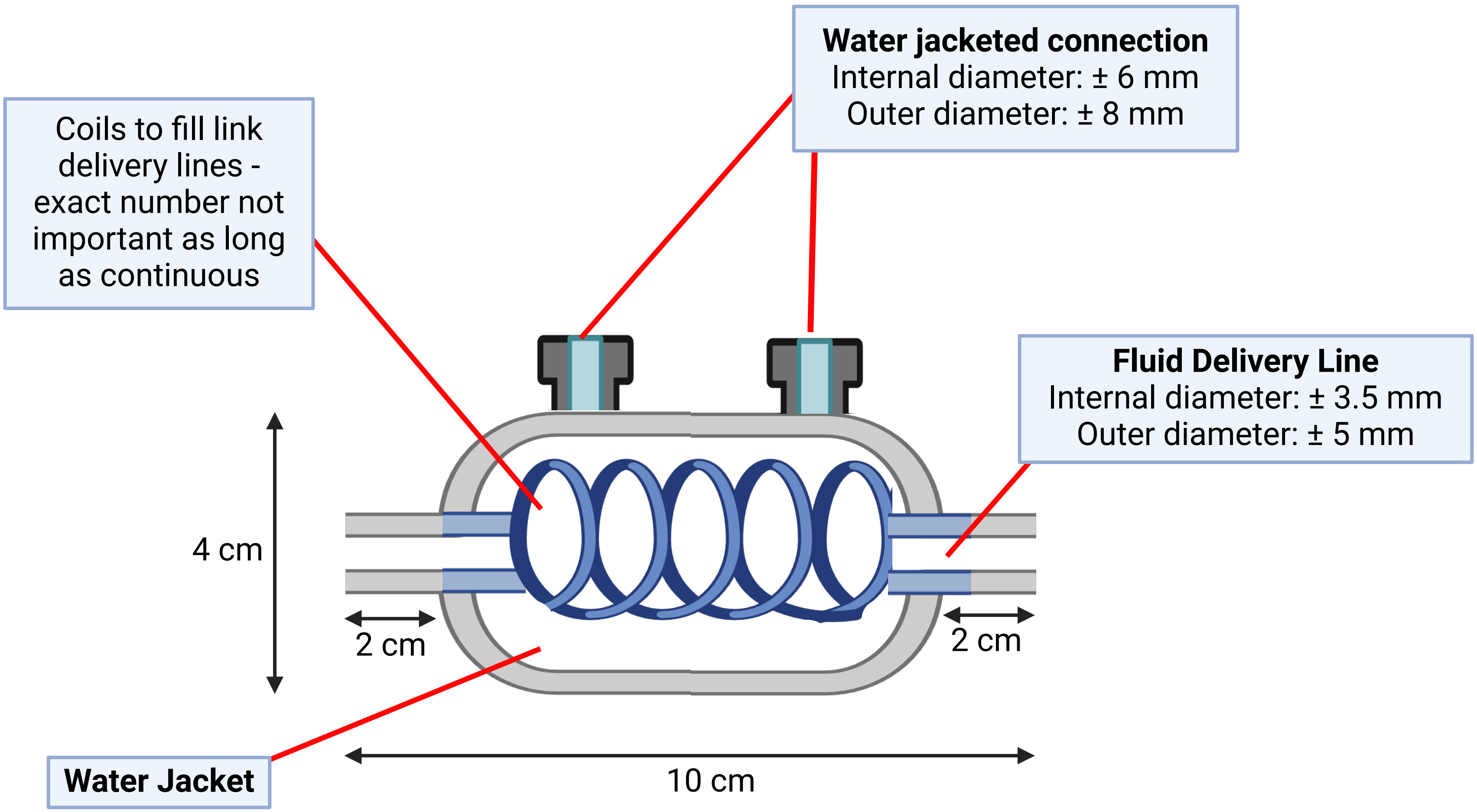

Supplementary Figure S2 Blueprint for a water jacketed spiral heat exchanger (not drawn to scale; related to Key Resources Table).

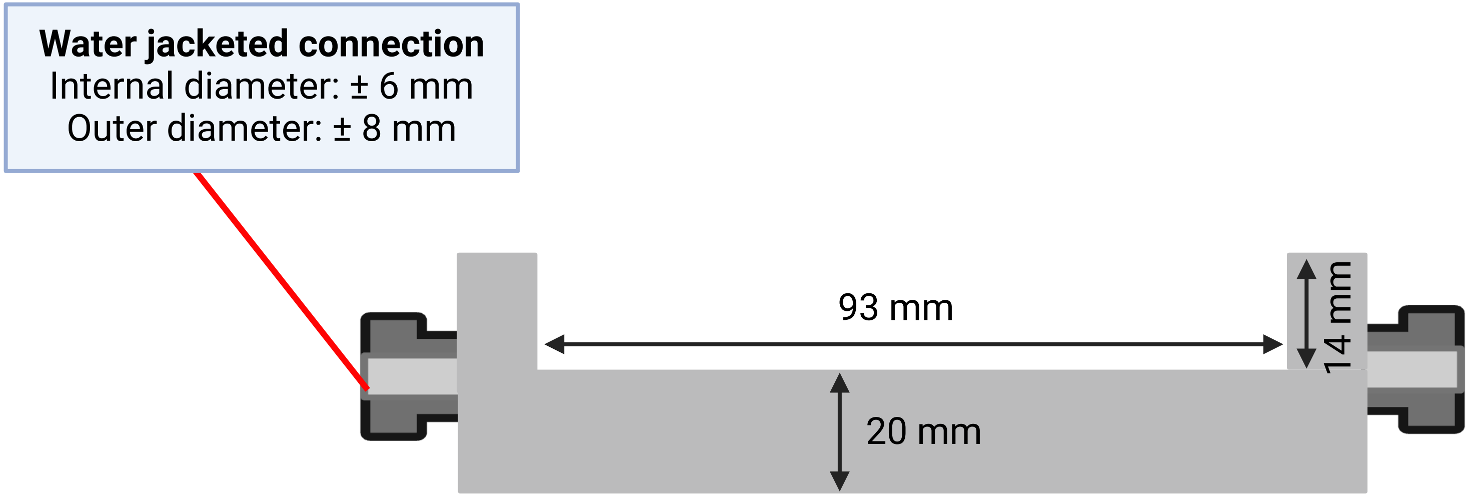

Supplementary Figure S3 Blueprint for a water jacketed petri dish warmer (not drawn to scale; related to Key Resources Table).
